# Efficacy of T-DM1 for leptomeningeal and brain metastases in a HER2 positive metastatic breast cancer patient: new directions for systemic therapy - a case report and literature review

**DOI:** 10.1186/s12885-018-3994-5

**Published:** 2018-01-25

**Authors:** Giuseppina Rosaria Rita Ricciardi, Alessandro Russo, Tindara Franchina, Silvia Schifano, Giampiero Mastroeni, Anna Santacaterina, Vincenzo Adamo

**Affiliations:** 1 Medical Oncology Unit, A.O. Papardo & Department of Human Pathology University of Messina, Messina, Italy; 2 Radiodiagnostic Unit, A.O. Papardo, Messina, Italy; 3 Radiation Oncology Unit, A.O. Papardo, Messina, Italy

**Keywords:** Trastuzumab Emtansine, T-DM1, Leptomeningeal metastases, Brain metastases, HER2-positive, Whole brain radiotherapy

## Abstract

**Background:**

Herein, we report a complete response after whole brain radiotherapy (WBRT) and concomitant T-DM1 in a patient with HER2-positive metastatic breast cancer (MBC) and extensive brain and leptomeningeal involvement.

**Case presentation:**

A 46 years old Caucasian woman with HER2-positive MBC and no baseline CNS involvement, started in August 2015 1^st^ line therapy with Pertuzumab-Trastuzumab-Docetaxel, with partial response. However, in April 2016 the patient eventually progressed with emergence of brain and leptomeningeal metastases. Hence, she started in May 2016 2^nd^ line therapy with T-DM1 and concomitant WBRT, with complete response (CR) after 3 courses of therapy, with complete resolution of neurological symptoms and no relevant toxicities. The CR is lasting over 13 months and the patient is out of corticosteroid use.

**Conclusions:**

To the best of our knowledge, this is the first case reporting interesting antitumor activity of T-DM1 and concomitant WBRT in both brain and leptomeningeal metastases, with a favorable safety profile and prolonged extracranial disease control. Further prospective studies should confirm these findings.

## Background

Human epidermal growth factor receptor 2 (HER2)-positive breast cancers (BCs) represent a distinct molecular subtype (~ 15–20% of all cases), defined by the overexpression of HER2 protein by immunohistochemistry (IHC) (IHC 3+) and/or amplification of HER2 gene by fluorescence in situ hybridization (FISH). Since the development of Trastuzumab, several different agents have been developed to target HER2 in BC, profoundly changing the course of this disease [1].

Brain metastases (BMs) represent a major issue in clinical practice, being associated with significant morbidity and often a dismal prognosis. BC is the 2^nd^ most common solid malignancy that metastasizes to the central nervous system (CNS), with the highest rates in HER2-positive patients. The incidence of BMs in HER2-positive BCs is growing as a consequence of the success of anti-HER2 targeted therapies, leading to a substantial survival gain. BMs represent a largely unmet medical need, with no targeted systemic options for brain metastases from breast cancer [2, 3].

In addition to BMs, CNS involvement may also be associated with leptomeningeal metastases (LMs), an unusual (incidence of ~ 5% in BC) but unfavorable complication commonly associated with a very dismal prognosis (3.5–3.8 months) [4, 5]. The incidence of LMs is growing, but effective and specific therapeutic strategies are lacking.

Trastuzumab emtansine (T-DM1) is an antibody-drug conjugate, incorporating the HER2–targeted antitumor properties of Trastuzumab with the cytotoxic activity of the microtubule-inhibitory agent DM1, which demonstrated a significant improvement in overall survival in Trastuzumab-pretreated metastatic breast cancer (MBC) patients compared to Lapatinib-Capecitabine [6] and treatment physician’s choice [7], respectively.

The retrospective analysis of the phase III trial EMILIA [8] and a few case report and case series [9–14] suggested a possible activity in patients with BMs. Scant data are available for the activity of anti-HER2 agents in patients with LMs, commonly excluded from most of randomized clinical trials.

Herein, we report a complete response after WBRT and concomitant T-DM1 in a patient with HER2-positive MBC and extensive brain and leptomeningeal involvement.

## Case presentation

**Fig. 1:**
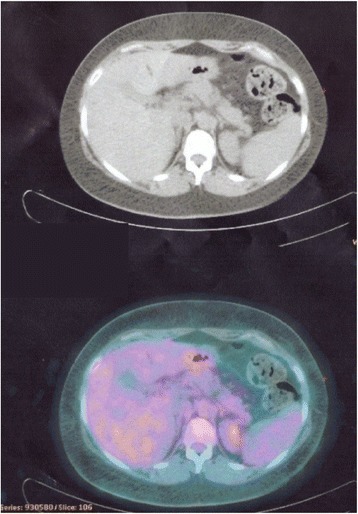
CT/PET scan showing CR in the liver lesions

In August 2015, a 46-year-old Caucasian woman referred to our Institution for a metastatic breast cancer. Three years before she had undergone a left quadrantectomy and ipsilateral axillary nodal dissection for a pT1c pN3 M0 infiltrating ductal carcinoma, grade 3, with Estrogen Receptor negative, Progesterone Receptor negative, MIB-1 60%, HER2 immunohistochemistry 3+. Then, she underwent adjuvant chemotherapy with doxorubicin and cyclophosphamide for four cycles followed by four courses of Docetaxel plus Trastuzumab and then Trastuzumab only for one year at standard doses. Thereafter, she started regular follow-up, negative for locoregional and distant recurrence, until July 2015, when an abdomen ultrasound showed multiple hypoechoic nodules in the liver (the largest with a maximum diameter of 22.3 × 20.7 mm), consistent with metastatic lesions. A positron emission tomography (PET) and a contrast-enhanced total body computed tomography (CT) scan confirmed the presence of multiple secondary lesions in the liver and multiple bilateral pulmonary and hilar lymph node metastases. According to the disease stage, biomolecular tumor characteristics, and clinical conditions (ECOG PS 0), in August 2015 she started 1^st^ line therapy with Pertuzumab plus Trastuzumab and Docetaxel every 3 weeks at standard doses. The CT scan after 3 courses of treatment showed a partial response with an almost complete disappearance and cystic transformation of both hepatic and pulmonary neoplastic lesions. Based on the tumor response, treatment was continued with the same regimen up to 6 cycles. In December 2015 the PET and CT restaging showed a radiological complete response (Fig. 1).

**Fig. 2:**
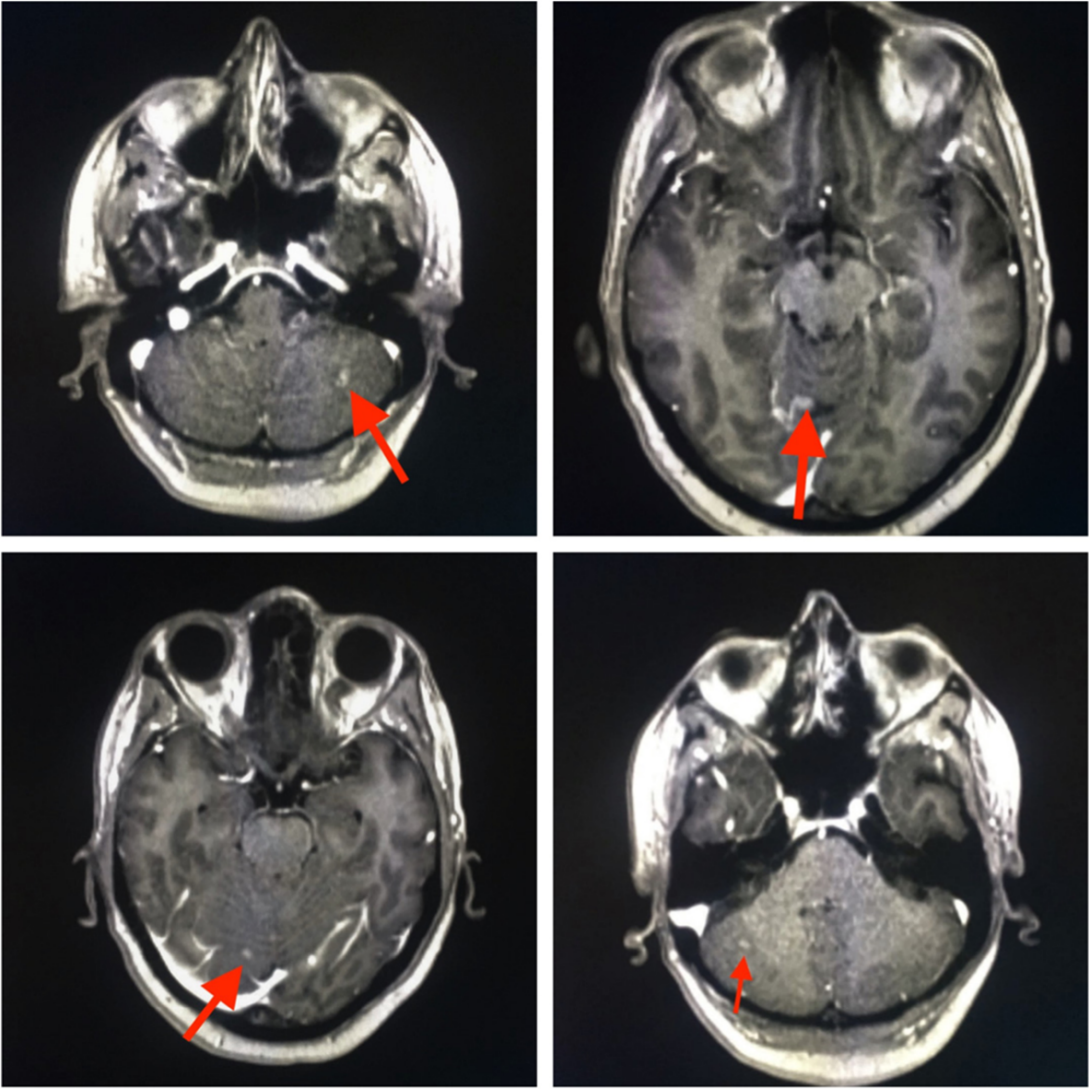
April 2016, Brain MRI showing multiple brain and leptomeningeal metastases

Hence, she reassumed the same regimen up to 8 courses and then she continued with only dual HER2-blockage for further 4 cycles. However, in April 2016 she started suffering from haze and headache. Brain CT and Magnetic Resonance Imaging (MRI) scans [Fig. 2] revealed the emergence of brain metastatic lesions and leptomeningeal metastases.

**Fig. 3:**
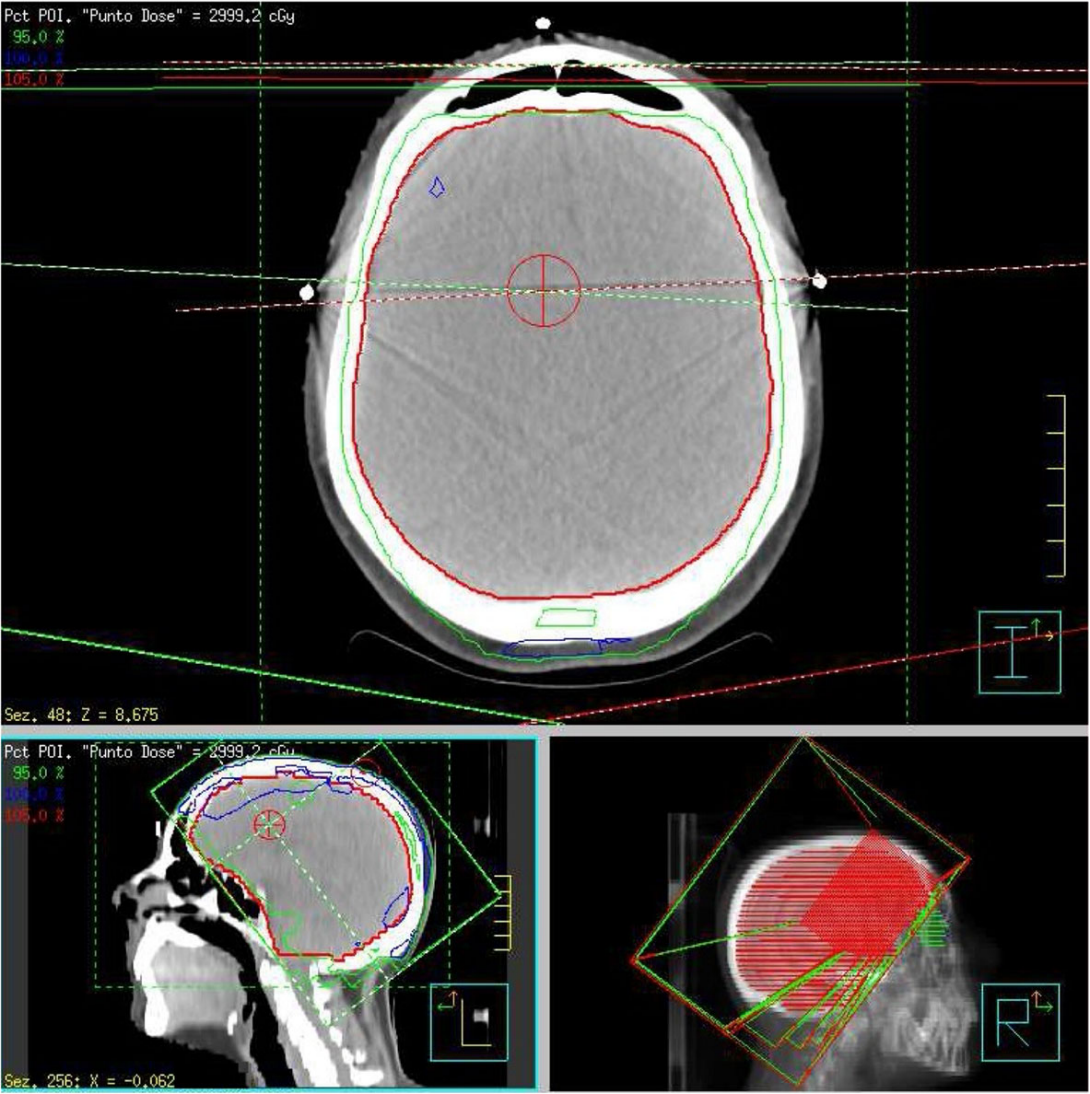
Treatment planning and delivery of Whole Brain Radiotherapy (WBRT)

Then, in May 2016 she started 2^nd^ line therapy with T-DM1 at 3.6 mg/kg every 3 weeks with concurrent Whole Brain Radiotherapy (WBRT) at total doses of 30 Gy in 10 fractions (Fig. 3).

**Fig. 4:**
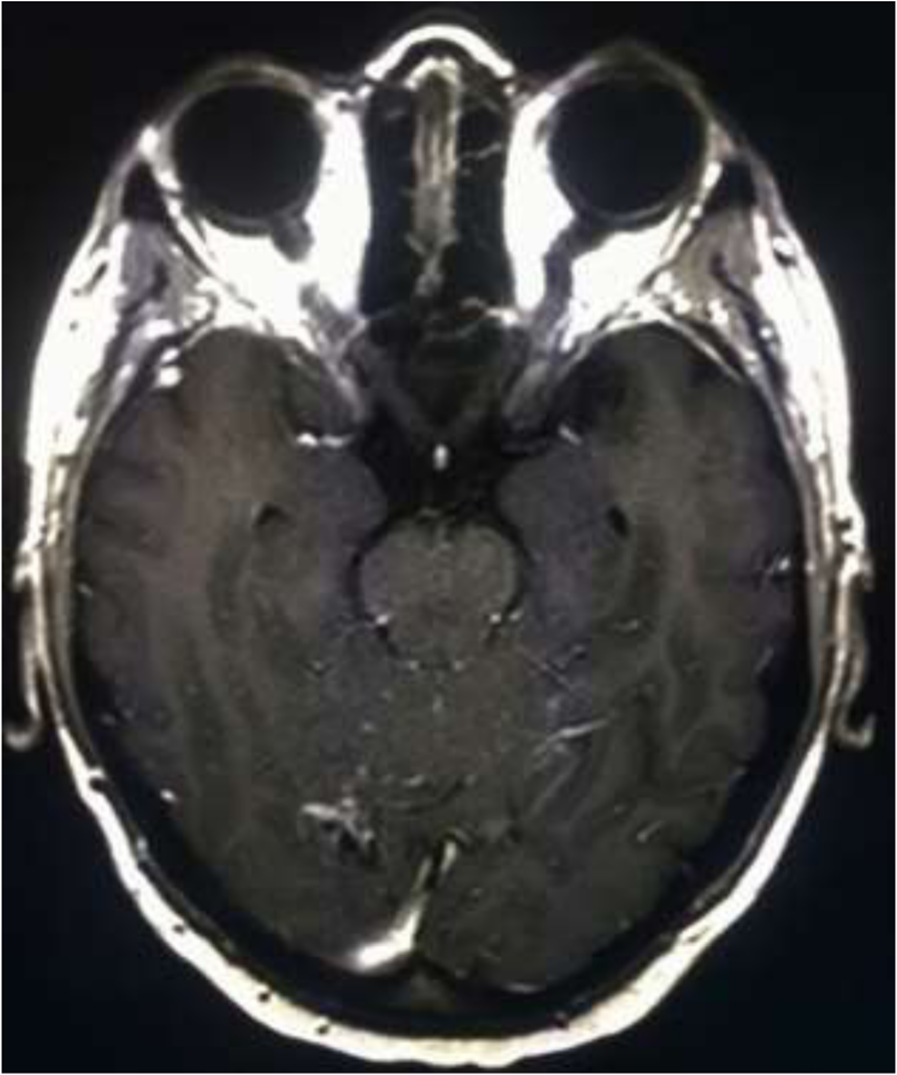
Brain MRI after 3 courses of T-DM1 therapy

A brain MRI after 3 courses of T-DM1 (Fig. 4) showed a complete response with a total disappearance of all CNS metastatic lesions and complete resolution of neurological symptoms.

**Fig. 5:**
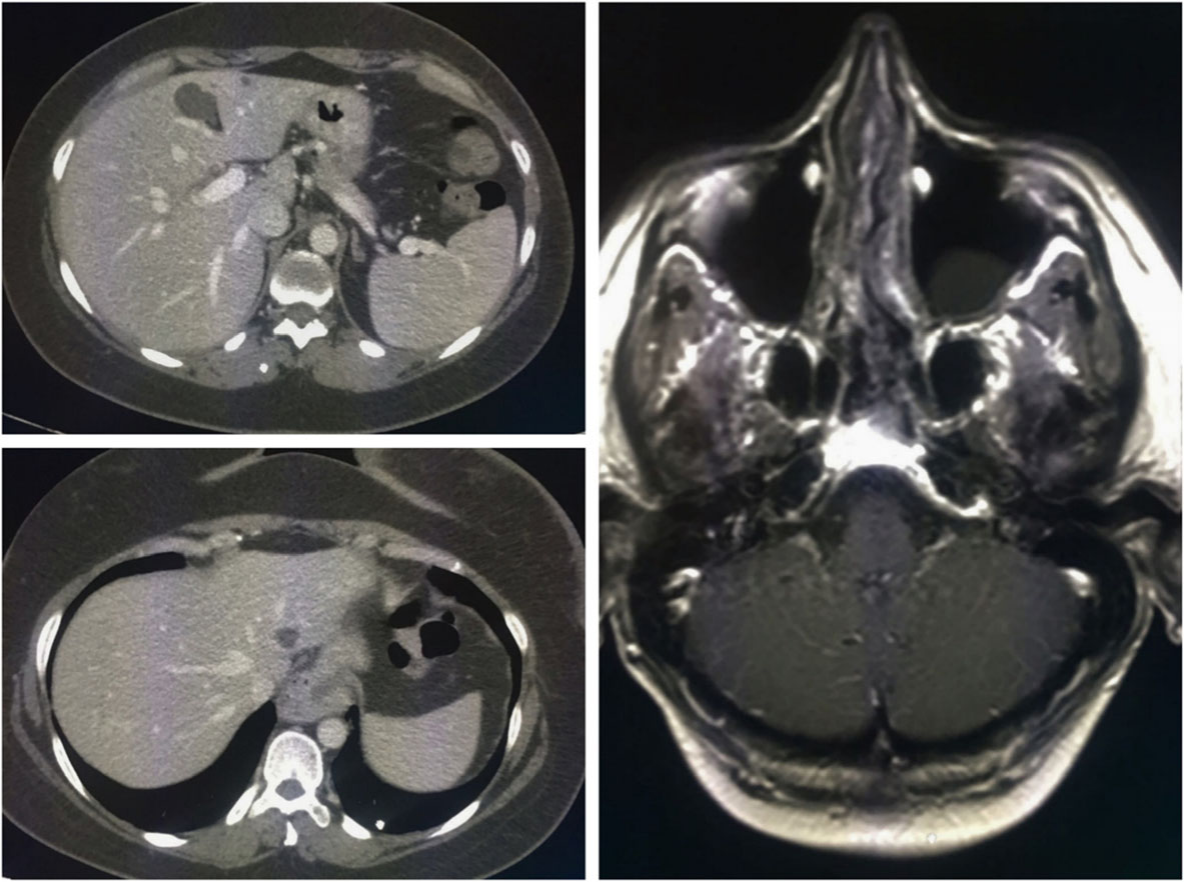
November 2016, the brain MRI confirms the CR in the CNS

**Fig. 6:**
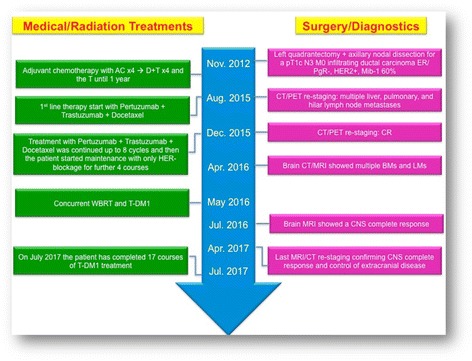
Timeline of interventions and outcomes

In April 2017, the brain MRI and thoracic-abdomen CT restaging (Fig. 5) after 14 cycles of T-DM1 confirmed the CNS complete response, with control of extracranial disease. To date, the patient has completed 17 courses of T-DM1 treatment (CNS PFS > 13 months), with no safety and neurological concerns without need of corticosteroid use (Fig. 6).

## Discussion and conclusions

Brain metastases (BMs) are a major cause of morbidity and mortality for patients with MBC and their incidence is growing. BC subtypes exhibit different propensity to metastasize to the CNS, with higher incidence of BMs in triple negative and HER2-positive breast cancers. Indeed, it is estimated that ~ 30–55% of patients with HER2-positive BCs will develop BMs during the course of their disease. Historically, the cornerstone for treatment of BMs was represented by loco-regional therapies, including whole brain radiotherapy (WBRT), stereotactic radiosurgery (SRS), and surgery. Given their limited blood-brain barrier (BBB) penetration and the exclusion of patients with BMs from the majority of randomized clinical trials, the role of systemic therapies in this setting is still unclear [15–17]. However, the development of targeted therapies has profoundly changed the natural history of molecularly selected cancer populations, including HER2-positive BCs, generating considerable interest in the activity of these agents against BMs [18].

CNS response rates with conventional chemotherapy ranges from 0% to 55% [16]. Monoclonal antibodies, such as Trastuzumab and Pertuzumab, have been traditionally thought not to cross the BBB, due to their relatively high molecular weights and the unlikely activation of ADCC process in the immuno-privileged brain microenvironment [9, 18]. However, preclinical evidences suggest that the integrity of BBB can be compromised in the presence of brain metastases and become increasingly permeable, due to loss of vascular pericyte coverage, dysregulation of tight junctions proteins, and increased VEGF secretion with subsequent increased synthesis of eNOS (endothelial Nitric Oxide Synthase) [19, 20]. Moreover, this increased permeability is not always homogeneous, with a small subset of BMs (~ 10%) having sufficient permeability to show a response to common cytotoxic agents [21]. Moreover, radiotherapy may further increase the BBB permeability [22].

Some preclinical data suggest that Trastuzumab may contribute to intracranial disease control, since it can cross an impaired BBB, especially in the presence of leptomeningeal involvement and prior radiation therapy [23, 24].

We previously reported a remarkable CNS activity with the nab-paclitaxel/Trastuzumab combination in a heavily pretreated HER2-positive BC patient with BMs [25].

Trastuzumab-containing regimens failed to prevent CNS failure [26], although retrospective studies have reported an improvement in OS for patients with BMs treated with Trastuzumab [27]. Therefore, it remains unclear whether the increased OS observed in these patients is attributable to a better extra-cranial disease control or to an intracranial activity of Trastuzumab [18, 28, 29]*.* Trastuzumab can be safely administrated during WBRT without increases in neurological toxicity [30].

Given its low molecular weight, Lapatinib, a dual EGFR/HER2 tyrosine kinase inhibitor, has been evaluated in BMs from BC. However, single agent Lapatinib reported only modest activity in HER2-positive BC patients with BMs progressing after WBRT (2.6–6% intracranial ORR) [31, 32], albeit the addition of Capecitabine may increase the CNS response rate up to 18–66% [33–37]. However, the use of Lapatinib-Capecitabine was not associated with lower incidence of CNS metastases compared with Trastuzumab-Capecitabine in the phase III trial CEREBEL [38].

Recently, the Pertuzumab-Trastuzumab-Docetaxel combination has been reported, in a subgroup analysis of the CLEOPATRA trial, to prolong the time to BMs emergence in HER2-positive BCs compared with Trastuzumab-Docetaxel-Placebo with a median time to development of CNS metastases as first site of disease progression of 11.9 months in the placebo arm vs. 15.0 months in the Pertuzumab arm (HR = 0.58, 95% CI 0.39–0.85, *P* = 0.0049), albeit the incidence of CNS metastases as first site of disease progression was similar in the two subgroups (12.6% in the placebo arm vs. 13.7% in the Pertuzumab arm) [39]. However, in the present case, the patient early recurred in the CNS with a BMs-free survival of only 9 months.

Recent preclinical work demonstrated that T-DM1 is active in murine models of BMs from HER2-positive BC, with a delayed growth of BMs, a longer survival benefit and a significant superior ADCC response in the brain microenvironment compared with Trastuzumab, due to the cytotoxic agent DM1 [40].

In a subgroup analysis of the EMILIA trial, T-DM1 was associated with a protective efficacy against BMs similar to that of Lapatinib-Capecitabine [8], two agents with known CNS penetration [41], with a similar incidence of CNS progression between the two subgroups, but with a longer OS with T-DM1 in both patients without BMs at baseline and those with treated, asymptomatic BMs [8].

A few case reports [9–11] and small case series [12–14] have suggested an intriguing intracranial activity of T-DM1 in patients with asymptomatic BMs with a 44–100% intracranial ORR [12, 14]. Moreover, de Vries et al. recently reported CNS activity also against symptomatic BMs [42].

No unexpected toxicities have emerged from these studies. However, given the relative limited number of patients evaluated in these studies no definitive conclusions can be drawn. Indeed, recently some authors pointed out the possible increase of neurological toxicities in patients with BMs treated with T-DM1 after SRS either as delayed [43] or rapid development of radiation necrosis [44]. Moreover, other authors have reported the concomitant use of radiotherapy and T-DM1 for the treatment of BMs [45, 46], but none of the patients included in those studies had leptomeningeal dissemination. The concomitant use of SRS and T-DM1 seems to increase the risk of radiation necrosis (50% in a small case series), a known complication of SRS, commonly observed in up to 34% of patients at 24 months and associated with a significant morbidity in 10–17% of patients [46].

The mechanisms of this interaction are largely unknown, albeit they may be related to an increased activity of T-DM1 after radiotherapy, since radiation is associated with HER2 upregulation in human breast cancer cell lines. Furthermore, T-DM1 may target injured glial cells, which are associated with upregulation of HER2 [44]. The synergistic activity of T-DM1 and radiotherapy combination is confirmed in the present case with a long, remarkable complete response in both parenchymal and leptomeningeal CNS metastases.

The safety profile of T-DM1 in association with radiotherapy (WBRT and SRS) either as sequential or concomitant therapy should be further evaluated in prospective studies. A Phase I study is evaluating different sequences of combined T-DM1 and WBRT (NCT02135159) and a phase II is being developed by the Translational Breast Cancer Research Consortium (TBCRC) to evaluate the activity of T-DM1 in BMs from HER2-positive BC [47]. The present case might suggest a good safety profile for combining WBRT and concomitant T-DM1, albeit further studies are needed to confirm these results.

Leptomeningeal metastases (LMs) represent an increasingly observed clinical scenario in HER2-positive BC due to the prolonged overall survival seen in these patients because of improvements in local and systemic therapies as well as supportive care. The incidence of LMs in breast cancer patients is ~ 5% and, despite major therapeutic breakthroughs in the last decade, it is still associated with a poorer prognosis (usually 2–4 months) than BMs [4, 48]. Treatment of LMs is a largely unmet medical need, commonly treated with local therapies, including radiotherapy and intrathecal/intraventricular therapy. No systemic agent has been proved effective in this setting, given the relative rarity of this clinical scenario and the exclusion of these patients from randomized clinical trials.

A recent large retrospective analysis of 318 patients with LMs from breast cancer, reported that survival, as for patients with BMs, is influenced by tumor subtype, since triple negative breast cancers (TNBCs) are associated with the worst OS, compared with HR+HER2- and with HR+/-HER2+. Interestingly, median OS of HER2-positive BCs with LMs improved in the last decade with the introduction of more effective therapeutic agents, such as Lapatinib, since the median survival was 3.3 months before 2005 and 7.0 months thereafter, suggesting that the use of systemic therapies with CNS activity may improve the outcome of these patients [4]. The role of T-DM1 is largely unknown in this subset.

To the best of our knowledge, this is the first case of a HER2-positive BC patient with LMs responding to T-DM1, with a long disease control in the CNS (intracranial PFS > 13 months), exceeding by far the short median OS commonly observed in these patients. The remarkable activity seen in the present case further supports the effect of T-DM1 in patients with CNS involvement, even in the presence of very difficult clinical scenarios, such as LMs. The impairment of the BBB caused by the presence of parenchymal and leptomeningeal metastases and prior radiotherapy may have favored the CNS penetration of this antibody-drug conjugate, enabling its antitumor activity even in a sanctuary site.

To the best of our knowledge, this is the first case reporting interesting antitumor activity of T-DM1 and concomitant WBRT in both brain and leptomeningeal metastases, with a favorable safety profile and prolonged extracranial disease control. Further prospective studies should confirm these findings.

The therapeutic landscape of HER2-positive BC patients with CNS involvement is rapidly evolving and we think that T-DM1 may play a key role in this scenario. The results of ongoing clinical trials with CNS-penetrant molecules, such as ONT-380 [49], are eagerly awaited and may add novel effective therapies in the therapeutic armamentarium of HER2-positive BCs metastasized to the CNS.

## Abbreviations

ADCC: Antibody-dependent cell-mediated cytotoxicity
BBB: Blood Brain Barrier
BC: Breast Cancer
BM: Brain Metastasis
CNS: Central Nervous System
CT: Computed Tomography
EGFR: Epidermal growth factor receptor
eNOS: Endothelial nitric oxide synthase
FISH: Fluorescent in situ hybridization
HER2: Human epidermal growth factor receptor 2
HR: Hormone Receptor
IHC: Immunohistochemistry
LM: Leptomeningeal Metastasis
MBC: Metastatic Breast Cancer
MRI: Magnetic Resonance Imaging
ORR: Overall Response Rate
PET: Positron Emission Tomography
PFS: Progression free survival
SRS: Stereotactic radiosurgery
T-DM1: Trastuzumab Emtansine
TNBC: Triple-negative breast cancer
WBRT: Whole Brain Radiation Therapy
